# Intrauterine and Postnatal Exposure to High Levels of Fluoride Is Associated with Motor Impairments, Oxidative Stress, and Morphological Damage in the Cerebellum of Offspring Rats

**DOI:** 10.3390/ijms23158556

**Published:** 2022-08-02

**Authors:** Deiweson Souza-Monteiro, Maria Karolina Martins Ferreira, Leonardo Oliveira Bittencourt, Walessa Alana Bragança Aragão, Igor Gonçalves de Oliveira, Cristiane Socorro Ferraz Maia, Marco Aurelio M. Freire, Fatemeh Vida Zohoori, Marília Afonso Rabelo Buzalaf, Rafael Rodrigues Lima

**Affiliations:** 1 Laboratory of Functional and Structural Biology, Biological Sciences Institute, Federal University of Pará, Belém 66075-110, Brazil; deiweson.monteiro@gmail.com (D.S.-M.); krolmarrtins93@gmail.com (M.K.M.F.); leo.bittencourt25@gmail.com (L.O.B.); walessa.aragao@gmail.com (W.A.B.A.); 2 Laboratory of Inflammation and Behavior Pharmacology, Pharmacy Faculty, Institute of Health Sciences, Federal University of Pará, Belém 66075-110, Brazil; igorgonoli1605@gmail.com (I.G.d.O.); crismaia@ufpa.br (C.S.F.M.); 3 Graduate Program in Health and Society, State University of Rio Grande do Norte, Mossoró 59610-210, Brazil; freire.m@gmail.com; 4 Centre for Public Health, School of Health and Life Sciences, Teesside University, Middlesbrough TS1 3BX, UK; v.zohoori@tees.ac.uk; 5 Department of Biological Sciences, Bauru School of Dentistry, University of São Paulo, Bauru 17012-901, Brazil; mbuzalaf@fob.usp.br

**Keywords:** fluoride, central nervous system, cerebellum, offspring, maternal exposure, oxidative stress, Purkinje cells, motor activity

## Abstract

Fluoride (F) is abundantly present on Earth and plays a beneficial role in human health. However, exposure to high doses of F can be a risk, mainly in endemic fluorosis regions. In light of this, we investigated the effects of F exposure during the intrauterine and postnatal periods of rats, in doses similar to those recommended in drinking water and the levels of F in regions with endemic fluorosis, on the offspring rats’ cerebellum. Pregnant rats were divided into three groups: control (received ultrapure water only), 10 mg F/L, and 50 mg F/L for a period of 42 days (21 days gestation and 21 days lactation). At the end of the lactation period, the male pups were evaluated by behavioral tests, morphological markers, and biochemistry assays. The results pointed out that 50 mg F/L exposure during the intrauterine and lactational period of rats is capable of promoting oxidative stress in the cerebellum with a decrease in Purkinje cell density and myelin basic protein compromise, which could be associated with functional motor impairments. In addition, although 10 mg F/L exposure promoted redox alterations, it did not affect other parameters evaluated, highlighting the safe use of F in low doses.

## 1. Introduction

Fluoride (F) is an element present in the Earth’s crust that is widely distributed in nature and found in several forms, such as calcium fluoride (CaF_2_) and sodium fluoride (NaF) [1]. This element has an important therapeutic role in preventing caries disease [2], as already been extensively demonstrated and consolidated in the literature [3]. Because of this, F has been incorporated in many oral health products, as well as in the water supply in many countries as a public health policy [4].

Although F has beneficial effects, excessive exposure may cause health concerns, promoting undesirable adverse effects [5]. Dental fluorosis is a disorder caused by problems related to systemic ingestion of F during dental formation [6]. Moreover, it can also impact bone (skeletal fluorosis) and the central nervous system (CNS) [1,7]. In this perspective, different regions around the world have reported public health problems associated with excessive F exposure [8,9]. Epidemiological studies in regions where F levels in the water are higher than recommended by the World Health Organization (WHO, Geneva, Switzerland; 0.5–1 ppm of F) revealed that children resident in these areas present reduced levels of intelligence quotient (IQ) when compared with children living in areas where this element is found in normal levels [10,11]. A recent systematic review with metanalysis from our group gathered such evidence from observational studies and showed that the clinical outcomes were more likely to be observed in people exposed to high concentrations of F [12].

Previous studies have already shown the ability of F to cross the blood-brain barrier (BBB) and trigger CNS impairments [13]. However, when it comes to F exposure during pregnancy, in addition to the BBB, the placenta barrier poses an important structure. The F can cross the placenta barrier, which is a permeable structure to water and elements such as calcium, iron, iodine, and F [14]. Furthermore, evidence supports that infants are also exposed to certain levels of F through breastmilk [15].

According to recent research [16], when pregnant rats were exposed to 10 mg F/L and 50 mg F/L until the end of the lactation period, the offspring rats showed significant compromise in the hippocampus, a cognitive-related CNS structure. In adult mice, it was observed that a high concentration (50 mg F/L) of F in drinking water was associated with motor deficits and biochemical impairments in the cerebellum [17]. However, the literature still lacks evidence regarding the effects of F exposure, at concentrations represented in daily human life, during the intrauterine and lactation periods on the motor-related structures of the CNS, such as the cerebellum of the offspring. 

In face of this socioenvironmental toxicology issue, the present work aimed to investigate the effects of F exposure during the intrauterine and postnatal periods of rats, in doses similar to those recommended in drinking water by the United States Environmental Protection Agency (EPA) to prevent enamel fluorosis and to the levels of F in regions with endemic fluorosis, evaluating motor coordination parameters. Moreover, this research aimed to unravel the oxidative biochemistry and morphological features of the F-exposed cerebellum of offspring rats for a better understanding of the effects of this exposure on brain development.

## 2. Results

### 2.1. The Influence of F on the Body Mass of Progenitor Rats Exposed to F during Gestational and Lactation Periods

As expected, all animals gained weight during the gestational period. After the experimental period (end of the lactation period), the mothers did not show a statistical difference in the body mass averages between the groups (*p* > 0.05).

### 2.2. Fluoride Exposure Triggered a Disbalance in the Oxidative State of the Cerebellum of Rats

**Figure 1:**
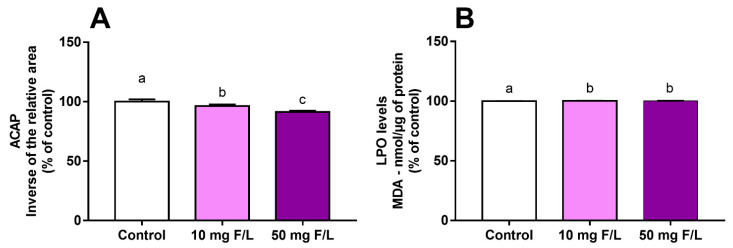
Effects on the cerebellum redox state of offspring after 42 days of fluoride exposure (10 mg F/L or 50 mg F/L, *n =* 5/group) during the gestational and lactation periods. Antioxidant capacity against peroxyl radicals (ACAP) levels as a percentage of control (**A**) and lipid peroxidation (LPO) levels measured by malonaldehyde (MDA) concentration as a percentage of control (**B**). The results are expressed as the mean ± SEM. One-way ANOVA and Tukey’s post hoc test, *p* < 0.05. Identical lowercase letters indicate the absence of a statistical difference.

The oxidative biochemistry assays revealed a decrease in antioxidant capacity against peroxyl radicals (ACAP) levels and increased levels of lipid peroxidation (LPO) in the cerebellum the offspring exposed to F at both low and high concentrations. The ACAP decreased in a dose-dependent manner (control: 100% ± 0.7896%; 10 mg F/L: 95.95% ± 0.5301%; 50 mg F/L: 91.33% ± 0.4383%; *p* < 0.0001) (Figure 1A). In addition, an increase in the LPO levels was evidenced in the exposed animals when compared to the control animals, while no difference was observed between the F groups (control: 100% ± 0.0249%; 10 mg F/L: 100.2% ± 0.2615%; 50 mg F/L: 100.2% ± 0.0328%; C vs. 10: adjusted *p* = 0.0003; C vs. 50: adjusted *p* = 0.0018; 10 vs. 50: adjusted *p* = 0.5536) (Figure 1B).

### 2.3. Prenatal and Postnatal Exposure to F at 50 Mg/L Was Associated with Cerebellar Morphology Damages in Rats

**Figure 2:**
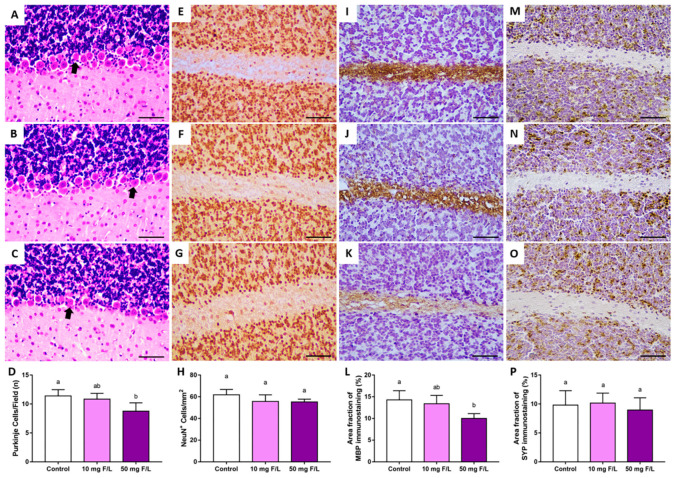
Effects on morphological aspects of the cerebellum of offspring after 42 days of fluoride exposure (10 mg F/L or 50 mg F/L, *n =* 5/group) during the gestational and lactation periods. Representative photomicrographs of sections stained in hematoxylin and eosin of the control (**A**), 10 mg F/L (**B**), and 50 mg F/L (**C**). Arrows indicate Purkinje cells. The results are expressed as the mean ± SEM of the number of Purkinje cells counted per field (one-way ANOVA and Tukey’s post hoc test, *p* < 0.05) (**D**). Representative photomicrographs of NeuN immunostaining (brown) sections of the control (**E**), 10 mg F/L (**F**), and 50 mg F/L (**G**). The results are expressed as the mean ± SEM of positive anti-NeuN cells counted per mm^2^ (one-way ANOVA and Tukey’s post hoc test, *p* < 0.05) (**H**). Representative photomicrographs of myelin basic protein (MBP) immunostaining (brown) sections of the control (**I**), 10 mg F/L (**J**), and 50 mg F/L (**K**). The results are expressed as the mean ± SEM of the area fraction percentage of positive anti-MBP immunostaining (Kruskal–Wallis and Dunn’s post hoc test, *p* < 0.05) (**L**). Representative photomicrographs of synaptophysin (SYP) immunostaining (brown) sections of the control (**M**), 10 mg F/L (**N**), and 50 mg F/L (**O)**. The results are expressed as the mean ± SEM of area fraction percentage of positive anti-SYP immunostaining (Kruskal–Wallis and Dunn’s post hoc test, *p* < 0.05) (**P**). Identical lowercase letters indicate the absence of a statistical difference. Scale bar = 5 µm.

The results showed that only 50 mg/L of F affected Purkinje cell density when compared to the control group. No significant differences were observed in the comparisons with the 10 mg F/L group (control: 11.38 ± 0.5543; 10 mg F/L: 10.83 ± 0.5; 50 mg F/L: 8.75 ± 0.7249; control vs. 10: adjusted *p* = 0.8037; control vs. 50: adjusted *p* = 0.0314; 10 vs. 50: adjusted *p* = 0.0848). Furthermore, exposure to 50 mg F/L also affected the area fraction of myelin basin protein (MBP) immunostaining when compared to the control, but no alteration was observed in the comparisons with the 10 mg/L F group (control median: 14.45 (interquartile range: 12.21–16.15); 10 mg F/L median: 12.61 (interquartile range: 12.1–15.4); 50 mg F/L median: 9.98 (interquartile range: 8.95–11); C vs. 10: adjusted *p* > 0.05; C vs. 50: adjusted *p* = 0.03; 10 vs. 50: adjusted *p* > 0.05). To analyze the mature neuron population, anti-neuronal nuclear protein (Anti-NeuN) immunostaining was performed. No significant difference was observed in all comparisons among the groups (control: 61.79 ± 4.974; 10 mg F/L: 55.72 ± 6.059; 50 mg F/L: 55.27 ± 2.529; *p* = 0.5079). In addition, synaptophysin immunostaining was also performed to analyze more tissue aspects of the cerebellum, but no statistical difference was observed (control median: 9.40 (interquartile range: 7.65–12.39); 10 mg F/L median: 10.67 (interquartile range: 8.35–11.43); 50 mg F/L median: 9.20 (interquartile range: 6.76–10.95); *p* = 0.81) (Figure 2).

### 2.4. F Exposure during Prenatal and Postnatal Periods Was Able to Promote Alterations in Rats’ Exploratory Behavior and Motor Coordination

**Figure 3:**
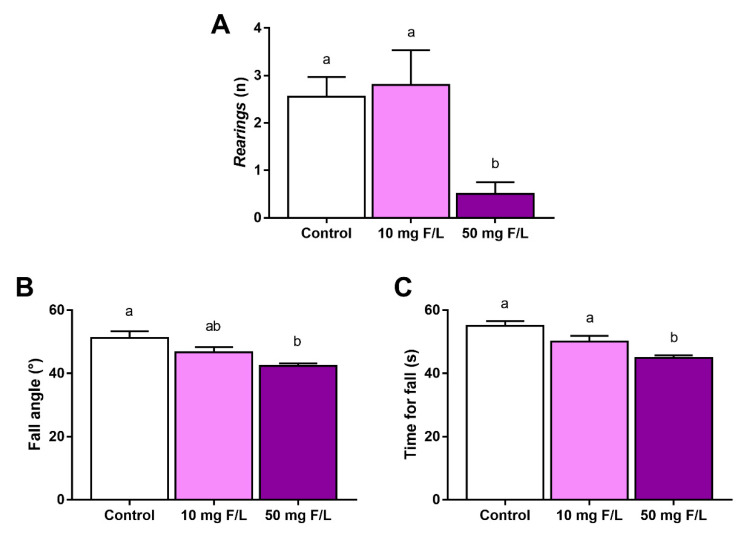
Effects on offspring behavior after 42 days of fluoride exposure (10 mg F/L or 50 mg F/L, *n =* 10/group) during the gestational and lactation periods. The number of *rearings* (**A**), fall angle in degrees (**B**), and time for fall in seconds (**C**). The results are expressed as the mean ± standard error of the mean (SEM). One-way ANOVA and Tukey’s post hoc test, *p* < 0.05. Identical lowercase letters indicate the absence of a statistical difference.

The number of *rearings* was reduced in the animals exposed to the higher dose of F when compared to the control animals and 10 mg F/L animals. The comparison between the control and 10 mg/L animals did not show a significant difference (control: 2.5 ± 0.412; 10 mg F/L: 2.8 ± 1.643; 50 mg F/L: 0.5 ± 0.2514; control vs. 10: *p* = 0.9264; control vs. 50: *p* = 0.0011; 10 vs. 50: *p* = 0.0026). The inclined plane test also revealed behavioral changes in the animals exposed to F. The test results showed a statistical difference in the fall angle of 50 mg F/L animals when compared to the control animals, while the 10 mg F/L animals did not show significant differences when compared to both control and 50 mg F/L animals (control: 51.25° ± 2.059°; 10 mg F/L: 46.67° ± 1.667°; 50 mg F/L: 42.33° ± 0.8262°; control vs. 10: adjusted *p* = 0.1058; control vs. 50: adjusted *p* = 0.0003; 10 vs. 50: adjusted *p* = 0.0723). The time of fall was reduced only in the 50 mg F/L animals in comparison to both control and 10 mg F/L groups, but no significant change was observed between control and 10 mg F/L groups (control: 55 ± 1.5 s; 10 mg F/L: 50 ± 1.8 s; 50 mg F/L: 44 ± 0.8 s; control vs. 10: adjusted *p* = 0.0535; control vs. 50: adjusted *p* = 0.0001; 10 vs. 50: adjusted *p* = 0.0231) (Figure 3).

## 3. Discussion

In the present work, we showed changes in the cerebellum of rats exposed to F during the intrauterine and lactational periods. These changes were related to oxidative stress in the organ and to functional damage such as motor locomotion and balance impairment. The F dose of 50 mg/L was also capable of promoting tissue alterations as those animals showed reduced Purkinje cell density and MBP immunostained area fraction, which may indicate high doses of F as a risk to the environmental health of endemic fluorosis regions.

F has well-established therapeutic actions against dental caries as it acts on the de/remineralization process; thus, its incorporation in oral health products and in the public water distribution system has been deeply discussed in many countries [14]. However, this is not the only source of F exposure; high levels of this ion are naturally found in many areas, such as in the lakes and soil of countries including India, China, and Mexico. Therefore, F exposure can occur naturally through the environment [18]. With this intense exposure to F in human life, many studies have proposed to evaluate the potential levels of F that could be dangerous to human health; notwithstanding, there is still no consensus on this topic, as low therapeutic doses have not yet been shown to trigger human health damage [19]. A recent meta-analysis indicated that, even though there is high heterogeneity in the available data, an association between IQ damage and exposure to F is only related to levels of F that exceed the recommended by public health institutions [12]. In our study, we used levels of F that could represent possible answers to this issue. The 10 mg/L dose of F is similar to the low dose of F used in drinking water for humans (approximately 2 mg F/L), and it was set by the EPA as a dose capable of preventing enamel fluorosis [20], while the 50 mg/L dose corresponds to high levels of F (10 mg/L of F) found in endemic fluorosis regions [21,22].

As F has been shown to be able to cross the placenta barrier, as well as the BBB, it has been associated with impairment in neurological development during prenatal and neonatal periods [13,23]. Moreover, low levels of F also are present in the breastmilk of mothers with a high intake of F [15], evidencing the early exposure of infants to F in utero and during postnatal periods. F levels in the blood have been reported in animals exposed to F during the gestational and lactational periods [16]. This study showed that this indirect exposure to F increased the F levels in the plasma of the offspring (nonexposed animals: 0.01 ± 0.009 μg/mL; 10 mg F/L: 0.03 ± 0.002 μg/mL; 50 mg F/L: 0.045 ± 0.005 μg/mL; *p* = 0.003). In addition, this bioavailability of F was associated with several changes in the hippocampus of the offspring, such as modulation of the proteomic profile and oxidative stress, with a dose-dependent response effect. Another study using a similar dosage of F also showed CNS impairments but in the cerebellum of adult rodents [24]. Such findings point out that F can trigger harmful effects on the CNS, which led us to investigate the effects of F on other regions, such as the cerebellum, of rats, exposed to these doses of F through maternal relationships (intrauterine and lactation). 

The control system for motion has a complex organization going from the basal ganglia controlling different command centers in the brainstem to the spinal cord and its executive circuits controlling muscles and, consequently, body movement [25]. The cerebellum contributes majorly to this process by fine-tuning locomotor movements and coordination [26]. It is responsible for predicting the sensory consequences of the movements, allowing compensatory adaptations, and its motoneurons act directly on the signal propagation and pathways that modify or correct ongoing locomotor activity [27]. The impairment of cerebellar-related functions (motor coordination, exploratory capacity, and animal balance) of adult animals exposed to 50 mg/L F was previously shown by our group after long-term exposure to F, and this motor refinement alteration was also associated with proteomic changes and oxidative stress [17]. 

F toxicity mechanisms are involved in many biological processes, such as protein inhibition [28], neuroinflammation [29], the release of free radicals [30], disruption of metal homeostasis [31], and tissue damage [32]. To better understand the possible effects of F on the cerebellum of the exposed offspring, the redox state of the organ was evaluated. Oxidative stress is defined as an imbalance due to the reduced activity of antioxidant mechanisms and increased levels of prooxidant molecules [33]. Our data showed higher levels of MDA in the F-exposed groups. MDA is a marker of polyunsaturated fatty-acid oxidation, which indicates lipid peroxidation [34]. In order to help the system affected by these damaged molecules, the antioxidant defense system tries to neutralize the action of these reactive species [35]. The increase in LPO and decrease in ACAP found in our data evidence that oxidative stress was possibly happening in the cerebellum of the rats exposed to F, suggesting that the organ’s enzymatic antioxidant compounds were failing to neutralize peroxyl radicals, thus enhancing possible damage. Although 10 mg/L F also showed a different redox status in comparison with the control group, these data were not sufficient to confirm damage, and other parameters should be investigated. Oxidative stress related to F exposure has been shown in many biological sites such as the plasma [36], jejunum [21], and kidneys [37]. Animals exposed to F had alterations in proteins related to cellular respiration, as well as tissue alterations, with apoptosis of liver cells observed [38].

From this perspective, we performed an evaluation of Purkinje cell density in the cerebellum of the rats. These cells are necessary for motor function and well-coordinated movements [39]. Purkinje cell apoptosis is related to movement disorders such as those observed in the spinocerebellar ataxia [40]. In our study, the animals exposed to 50 mg/L during the prenatal and neonatal period showed reduced Purkinje cell density, while the animals exposed to 10 mg/L did not show alterations in comparison with the control group. This indicates that the higher dose not only disturbed the redox status but also affected cerebellar cell density, probably resulting in harmful functional effects.

Furthermore, we also investigated other histological parameters such as the mature neuron population, synaptophysin, and MBP. Mature neurons and synaptic vesicles are important to maintain cerebellar homeostasis as they are responsible for the physiological transmission, communication, and execution of movements [41,42], but no alteration was observed in these parameters. However, MBP downregulation was observed in the 50 mg/L F group. Myelin is fundamental to the cerebellum as it plays an important role in the speed and propagation of nervous impulses on the axon [43]. Together with the Purkinje cell apoptosis observed in the 50 mg/L F group, this downregulation of MBP may have compromised functional motor activity.

To investigate the possible functional damage caused by the alteration discussed previously, the performance of the offspring exposed to F was evaluated. The parameters observed were related mainly to vertical locomotion and equilibrium. Our data showed that the high dose of F was capable of affecting the animals’ capacity for locomotion, as the number of *rearings* was reduced and the animals also showed poorer performance in the inclined plane test when compared to the control group (Figure 3). These behavioral impairments are associated with muscle weakness, tremors, and loss of balance [44,45]; hence, our findings are suggestive that F at doses ≥50 mg/L is capable of affecting the motor coordination and postural stability of animals. 

## 4. Materials and Methods

### 4.1. Ethics Statement and Experimental Groups

Albino Wistar pregnant rats (*Rattus norvegicus*) weighing 150–200 g (*n =* 9, 90 days old) were placed in plastic cages appropriate to the species following the Guide for the Care and Use of Laboratory Animals [46], under the license of the Ethics Committee with the use of Experimental Animals from the Federal University of Pará (protocol number 5316261120). The animals were randomly divided into three groups (*n =* 3/group); the control group received ultrapure water only, while one group received ultrapure water with 10 mg/L F (from NaF) and another group received ultrapure water with 50 mg/L F. The drinking water was renewed every 3 days. All groups received pelleted food (Presence, Neovia, Brazil) and ultrapure water ad libitum; the room was under standard conditions with a 12 h light/dark cycle and controlled temperature (25 °C).

**Figure 4:**
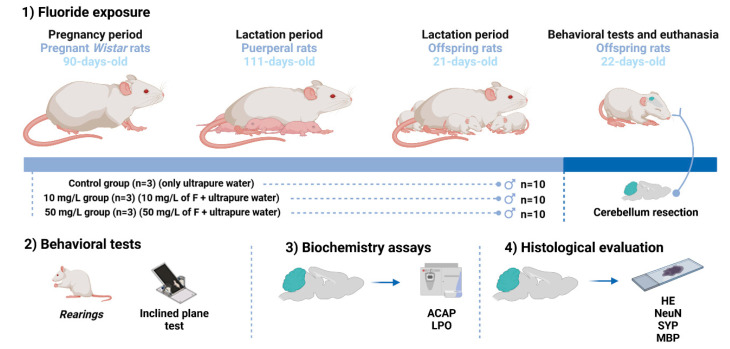
Methodological steps of the study. Experimental groups, fluoride exposure and cerebellum resection (**1**). Behavioral tests: Number of rearing and Inclined plane test (**2**). Histological evaluation: Hematoxylin & Eosin (HE) staining, Anti-NeuN immunostaining, Anti-Synaptophysin (SYP) immunostaining and Anti-Myelin basic protein (MBP) immunostaining (**3**). Biochemistry assays: Antioxidant capacity against peroxyl radicals (ACAP) and Lipid peroxidation (LPO) (**4**).

The 10 mg/L and 50 mg/L doses of F are similar to the F levels on artificially fluoridated drinking water and to the F levels of regions with endemic fluorosis, respectively, adapted to the rodents’ metabolism [4,22]. The gestational period lasted 21 days, then the offspring were conceived and the lactation period started and lasted 21 more days. The F exposure was made during both gestation (after the vaginal tampon identification) and lactation period, for a total of 42 days. A previous study using the same F exposure (i.e., both doses and gestational-lactation period) showed increased F levels in the plasma of offspring in groups exposed to 10 mg F/L (0.03  ±  0.002 μg/mL) and 50 mg F/L (0.045  ±  0.005 μg/mL) in comparison to a non-exposed group (0.01  ±  0.009 μg/mL; *p* = 0.003) [16]. When the lactation period ended, the offspring were divided by sex and the male animals were used. Thus, each experimental group had 10 male pups. All methodological steps are summarized in Figure 4.

### 4.2. Behavioral Tests

After the end of the lactation period, behavioral tests were conducted with the offspring. All tests were performed in a room with attenuation of noise levels and low light intensity in order to prevent stress on the animals. One hour before the start of the experiments, the animals were brought to the testing room for acclimatization and habituation to the environment. Firstly, each animal (*n =* 10/group) was placed in the center of an acrylic arena (100 × 100 × 40 cm; Insight, São Paulo, Brazil) and observed for 5 min. The number of *rearings* (i.e., the number of times the animal stood while supported on the hind legs) was analyzed to evaluate vertical equilibrium and exploration [47].

The animals were also submitted to the inclined plane test. In this test, the animal’s ability to maintain postural stability was evaluated. Animals with impaired motor coordination and equilibrium are unable to perform descent and ascent movements on a bar with a slope greater than 45°. According to the protocol previously described [48], the animals were submitted to a horizontal flat platform (Insight, São Paulo, Brazil), in which the angle of inclination was increased by 5° until the animal was able to maintain its position for up to 5 s. The latency until the fall and the final angle supported by the animal were counted in five consecutive trials (intertrial interval of 60 s). The final score was the average angle calculated across all trials.

### 4.3. Biological Sample Resection

After the behavioral tests, the animals of each group (*n =* 10) were anesthetized with a solution of ketamine hydrochloride (90 mg/kg, 10%) (Rompum, Bayer Saúde Animal, Brazil) and xylazine hydrochloride (2 mg/kg, 2%) (Dopalen, Agribands, Brazil). After complete abolishment of corneal and limb reflexes, five animals from each group were perfused for histological analysis. The remaining five animals from each group were submitted to a craniotomy for cerebellum resection. The organ was immediately frozen in liquid nitrogen and stored in an ultra-freezer at −80 °C for further biochemistry assays. 

### 4.4. Perfusion and Histological Analysis

Five animals from each group were deeply anesthetized and submitted to perfusion with 0.9% cold saline solution, heparin (1%), and warm 4% paraformaldehyde-buffered solution (0.2 M). The cerebellum was removed for histological procedures, post-fixed in Bouin solution for 6 h, dehydrated in increasing solutions of ethanol (70%, 80%, 90%, absolute I, and absolute II), diaphanized in xylol I, xylol II, and embedded in Paraplast (McCormick Scientific, Baltimore, MD, USA). Using a microtome (Leica Biosystems, RM2125 RTS, Nussloch, Germany), sections of 5 μm thickness were obtained.

#### 4.4.1. Purkinje Cell Counting

For evaluation of Purkinje cell density, the sections obtained by microtomy were stained by routine hematoxylin–eosin (HE) and then analyzed by light microscopy (Nikon Eclipse E200, Tokyo, Japan), using a grid corresponding to an area of 0.0625 mm^2^ coupled to one of the eyepieces, using an objective lens with 40× magnification. At least three fields in the cerebellum per section and one section per animal of each group were analyzed to count the number of Purkinje neurons per field [49].

#### 4.4.2. Immunohistochemical Assays

For immunohistochemical analyses, the slides with sections were dewaxed, rehydrated by heat in xylene and hydroalcoholic solutions, and immersed in 0.1 M phosphate-buffered saline (PBS) for 3 min before incubation in citrate buffer at 65 °C for 25 min. After antigen retrieval, the sections were immersed in PBS for 10 min and immersed in methanol–hydrogen peroxide solution (3:100, *v*/*v*) for endogenous peroxidase blockage. Anti-NeuN (1:100, Chemicon International, Inc., Temecula, CA, USA), anti-myelin basic protein (1:250, Promega Corp., Madison, WI, USA), and anti-synaptophysin (1:1000, Wako Chemicals Ltd., Osaka, Japan) antibodies for immunolabeling of mature neurons, as well as myelin basic protein (MBP) (1:100; Chemicon International, Inc., Temecula, CA, USA) and synaptic vesicles, as previously established by our group, were applied [42]. The protocol included 3,3′-diaminobenzidine solution in 0.1 M PBS; sections immunolabeled with anti-MBP and anti-synaptophysin were counterstained with Mayer’s hematoxylin. All slides were dehydrated and mounted on a coverslip with Entellan^®^ (Merck, Darmstadt, Germany) [50].

The positive cells for anti-NeuN immunostaining were analyzed in photomicrographs obtained with the DS-Fi3 microscopic camera attached to the microscope Nikon Eclipse CiH550s (Tokyo, Japan) using an objective lens with 40× magnification, evaluating the density of anti-NeuN^+^ cells in three photomicrographs of two sections per animal. For analyses of anti-MBP and anti-synaptophysin immunostaining, we first obtained the photomicrographs using the DS-Fi3 microscopic camera attached to the above-cited microscope Nikon Eclipse CiH550s using an objective lens with 40× magnification. Then, the photomicrographs were analyzed using ImageJ software with the Color Deconvolution plugin according to previous studies from our group [42,51], which extracted the fraction of area immunolabeled by the antibodies and revealed by DAB. The results were expressed as the fraction of area (%) immunolabeled in comparison to the total area of the section captured by the camera system.

### 4.5. Oxidative Biochemistry Assays

As pretreatment for the biochemical assays, the cerebellum samples were thawed and resuspended in a Tris-HCl solution (20 mM, pH 7.4) for sonic disintegration (~1 g/mL). The supernatants were used in the antioxidant capacity against peroxyl radicals (ACAP) assay and lipid peroxidation (LPO) assay.

#### 4.5.1. Antioxidant Capacity against Peroxyl Radicals

ACAP was analyzed using the reactive oxygen species (ROS) quantitation produced by the equally concentrated samples (2.5 µg proteins/µL) after being exposed to a peroxyl radical generator [52]. Peroxyl radicals were produced by the thermal (35 °C) decomposition of 2,2′-azobis(2-methylpropionamidine) dihydrochloride (ABAP; 4 mM; Sigma-Aldrich, St. Louis, MI, USA). For ROS determination, the compound 2′,7′-dichlorofluorescein diacetate (H2DCF-DA, Invitrogen™, Waltham, MA, USA) was used at a final concentration of 40 nM. The readings were carried out in a fluorescence microplate reader (Victor X3, Perkin Elmer, Waltham, MA, USA) every 5 min for 1 h. The relative difference between the ROS area with and without ABAP was considered as a measure of antioxidant capacity. The results were transformed to the inverse of the relative area and expressed as a percentage of the control.

#### 4.5.2. Lipid Peroxidation

MDA levels were used as an indicator to determine LPO levels in the samples [52]. The lysates were centrifuged at 3512× *g* for 10 min at 4 °C after sonic homogenization. MDA supernatants and standard solutions were then incubated at 45 °C for 40 min with a 1:4 solution of methanesulfonic acid and *N*-methyl-2-phenylindole (10.3 mM) diluted in methanol (1:3), followed by spectrophotometric reading (570 nm). The results were expressed in nanomoles per microgram (nmol/µg) of protein and graphed as a percentage of the control. Protein quantification was performed using the Bradford method [53].

### 4.6. Statistical Analyses

The data were plotted using GraphPad Prism 7.0 (GraphPad Software Inc., La Jolla, CA, USA). For the analysis of the Gaussian distribution of our data, we performed the Shapiro–Wilk normality test. The analyses of body weight gain were carried out using a two-way ANOVA repeated measure followed by Tukey’s post hoc test with *p* < 0.05. The normal data were submitted to the one-way ANOVA test, followed by Tukey’s test to perform comparisons between the groups, considering the statistical significance level of *p* < 0.05. The nonparametric data were submitted to the Kruskal–Wallis test, followed by Dunn’s test to perform a comparison between the groups considering *p* < 0.05. The biochemistry assay data were expressed as a percentage of control. Results were expressed as the mean ± standard error of the mean. The test power was calculated using the difference between the group averages with the OpenEpi software (Version 2.3.1, Emory University, Atlanta, GA, USA), considering a type I error of 5% and a power of 80%. The description of all analysis values can be found in Table S1.

## 5. Conclusions

Our study demonstrated that exposure of rats to 50 mg/L F during the intrauterine and lactational periods is capable of promoting oxidative stress in the cerebellum with Purkinje cell apoptosis and MBP downregulation, which could culminate in functional motor impairments. Our data also showed that, although 10 mg/L F promoted redox status alterations, it did not affect the histological and functional parameters evaluated, highlighting the safety of low doses of F, such as those present in artificially fluoridated water. Further investigations including molecular and other oxidative stress markers are necessary in order to better elucidate the effects of F, mainly related to its dose and period of exposure.

## Data Availability

All data are available within the article.

## Supplementary Materials

The following supporting information can be downloaded at https://www.mdpi.com/article/10.3390/ijms23158556/s1.
