# Supplementary material for: Intrauterine and Postnatal Exposure to High Levels of Fluoride Is Associated with Motor Impairments, Oxidative Stress, and Morphological Damage in the Cerebellum of Offspring Rats
Source: Int J Mol Sci. 2022 Aug 2;23(15):8556. doi: 10.3390/ijms23158556 (PMC9369436; doi:10.3390/ijms23158556)
Supplement: Supplementary file 1 [file ijms-23-08556-s001.zip › ijms-1774548-supplementary.pdf]

Table S1. Description of all analysis values of the study.

Analysis	Description of unit	Mean (Control)	SD (Control)	SEM (Control)	Mean (10 mg F/L)	SD (10 mg F/L)	SEM (10 mg F/L)	Mean (50 mg F/L)	SD (50 mg F/L)	SEM (50 mg F/L)	Power (1-β err prob)
**ACAP**	% of control	100	1.934	0.789	95.95	1.185	0.530	91.33	1.074	0.438	Control *vs* 10: 97.9%<br>Control *vs* 50: 100%<br>10 *vs* 50: 100%
**LPO**	% of control	100	0.055	0.249	100.2	0.058	0.026	100.2	0.073	0.032	Control *vs* 10: 99.99%<br>Control *vs* 50: 99.83%<br>10 *vs* 50: 1.07%
**Purkinje cells density**	Number per field	11.38	1.109	0.554	10.83	1	0.5	8.75	1.45	0.724	Control *vs* 10: 12.76%<br>Control *vs* 50: 89.65%<br>10 *vs* 50: 75.2%
**NeuN**	Cells/mm^2	61.79	11.12	4.974	55.72	12.12	6.059	55.27	6.194	2.529	Control *vs* 10: 12.79%<br>Control *vs* 50: 20.77%<br>10 *vs* 50: 1.84%
**MBP**	Area fraction (%)	14.27	2.128	1.064	13.37	1.938	0.969	10.01	1.104	0.551	Control *vs* 10: 10.3%<br>Control *vs* 50: 97.8%<br>10 *vs* 50: 92.05%
**SYP**	Area Fraction (%)	9.816	2.492	1.246	10.15	1.724	0.862	8.973	2.102	1.214	Control *vs* 10: 3.72%<br>Control *vs* 50: 8.20%<br>10 *vs* 50: 16.06%
**Rearings**	Number	2.556	1.236	0.412	2.8	1.643	0.734	0.5	0.940	0.251	Control *vs* 10: 7.46%<br>Control *vs* 50: 100%<br>10 *vs* 50: 99.97%
**Fall angle**	°	51.25	5.825	2.059	46.67	5	1.667	42.33	3.2	0.826	Control *vs* 10: 76.06%<br>Control *vs* 50: 100%<br>10 *vs* 50: 90.49%
**Time for fall**	Seconds	55	4.243	1.5	50	5.612	1.871	44.93	3.025	0.808	Control *vs* 10: 88.85%<br>Control *vs* 50: 100%<br>10 *vs* 50: 94.48%

Table S1: Oxidative biochemistry assays (ACAP: Antioxidant Capacity Against Peroxyl Radicals and LPO: Lipid Peroxidation), Histological analysis (Purkinje cells density, Anti-NeuN, Anti-Myelin Basic Protein and Anti-Synaptophysin) and Behavior tests (Rearings and Inclined Plane [Fall angle and Time for fall]) values of the experimental study. Results are expressed as mean, SD: Standard deviation, SEM: Standard error of mean and Test Power (1-β error probability).
